# Immune correlates analysis of the mRNA-1273 COVID-19 vaccine efficacy clinical trial

**DOI:** 10.1126/science.abm3425

**Published:** 2021-11-23

**Authors:** Peter B. Gilbert, David C. Montefiori, Adrian B. McDermott, Youyi Fong, David Benkeser, Weiping Deng, Honghong Zhou, Christopher R. Houchens, Karen Martins, Lakshmi Jayashankar, Flora Castellino, Britta Flach, Bob C. Lin, Sarah O’Connell, Charlene McDanal, Amanda Eaton, Marcella Sarzotti-Kelsoe, Yiwen Lu, Chenchen Yu, Bhavesh Borate, Lars W. P. van der Laan, Nima S. Hejazi, Chuong Huynh, Jacqueline Miller, Hana M. El Sahly, Lindsey R. Baden, Mira Baron, Luis De La Cruz, Cynthia Gay, Spyros Kalams, Colleen F. Kelley, Michele P. Andrasik, James G. Kublin, Lawrence Corey, Kathleen M. Neuzil, Lindsay N. Carpp, Rolando Pajon, Dean Follmann, Ruben O. Donis, Richard A. Koup, Immune Assays Team, Moderna, Inc. Team, Coronavirus Vaccine Prevention Network (CoVPN)/Coronavirus Efficacy (COVE) Team, United States Government (USG)/CoVPN Biostatistics Team

**Affiliations:** 1 Vaccine and Infectious Disease Division, Fred Hutchinson Cancer Research Center, Seattle, WA, USA.; 2 Public Health Sciences Division, Fred Hutchinson Cancer Research Center, Seattle, WA, USA.; 3 Department of Biostatistics, University of Washington, Seattle, WA, USA.; 4 Department of Surgery and Duke Human Vaccine Institute, Duke University Medical Center, Durham, NC, USA.; 5 Vaccine Research Center, National Institute of Allergy and Infectious Diseases, National Institutes of Health, Bethesda, MD, USA.; 6 Department of Biostatistics and Bioinformatics, Rollins School of Public Health, Emory University, Atlanta, GA, USA.; 7 Moderna, Inc., Cambridge, MA, USA.; 8 Biomedical Advanced Research and Development Authority, Washington, DC, USA.; 9 Division of Biostatistics, School of Public Health, University of California Berkeley, Berkeley, CA, USA.; 10 Department of Molecular Virology and Microbiology, Baylor College of Medicine, Houston, TX, USA.; 11 Brigham and Women’s Hospital, Boston, MA, USA.; 12 Palm Beach Research Center, West Palm Beach, FL, USA.; 13 Keystone Vitalink Research, Greenville, SC, USA.; 14 Department of Medicine, Division of Infectious Diseases, UNC HIV Cure Center, University of North Carolina at Chapel Hill School of Medicine, Chapel Hill, NC, USA.; 15 Division of Infectious Diseases, Department of Medicine, Vanderbilt University Medical Center, Nashville, TN, USA.; 16 Division of Infectious Diseases, Department of Medicine, Emory University School of Medicine and the Grady Health System, Atlanta, GA, USA.; 17 Department of Laboratory Medicine and Pathology, University of Washington, Seattle, WA, USA.; 18 Center for Vaccine Development and Global Health, University of Maryland School of Medicine, Baltimore, MD, USA.; 19 Biostatistics Research Branch, National Institute of Allergy and Infectious Diseases, National Institutes of Health, Bethesda, MD, USA.

## Abstract

In the coronavirus efficacy (COVE) phase 3 clinical trial, vaccine recipients were assessed for neutralizing and binding antibodies as correlates of risk for COVID-19 disease and as correlates of protection. These immune markers were measured at the time of second vaccination and 4 weeks later, with values reported in standardized World Health Organization international units. All markers were inversely associated with COVID-19 risk and directly associated with vaccine efficacy. Vaccine recipients with postvaccination 50% neutralization titers 10, 100, and 1000 had estimated vaccine efficacies of 78% (95% confidence interval, 54 to 89%), 91% (87 to 94%), and 96% (94 to 98%), respectively. These results help define immune marker correlates of protection and may guide approval decisions for messenger RNA (mRNA) COVID-19 vaccines and other COVID-19 vaccines.

## Antibody levels predict vaccine efficacy

Symptomatic COVID-19 infection can be prevented by severe acute respiratory syndrome coronavirus 2 (SARS-CoV-2) vaccines. A “correlate of protection” is a molecular biomarker to measure how much immunity is needed to fight infection and is key for successful global immunization programs. Gilbert *et al*. determined that antibodies are the correlate of protection in vaccinated individuals enrolled in the Moderna COVE phase 3 clinical trial (see the Perspective by Openshaw). By measuring binding and neutralizing antibodies against the viral spike protein, the authors found that the levels of both antibodies correlated with the degree of vaccine efficacy. The higher the antibody level, the greater the protection afforded by the messenger RNA (mRNA) vaccine. Antibody levels that predict mRNA vaccine efficacy can therefore be used to guide vaccine regimen modifications and support regulatory approvals for a broader spectrum of the population. —PNK

## Teaser

SARS-CoV-2 binding and neutralizing antibodies correlate with the degree of vaccine efficacy and protection for the Moderna mRNA COVID-19 vaccine.

On the basis of their demonstrated efficacy to prevent COVID-19 in phase 3 clinical trials, to date, seven COVID-19 vaccines have been granted an emergency use listing by the World Health Organization (WHO) (*1*), three have been granted an emergency use authorization (EUA) by the US Food and Drug Administration (FDA) (*2*), and one has been formally approved by the FDA (*3*). However, the manufacturing challenges posed by the global demand for doses, the need for affordable and accessible options that are safe and effective in diverse populations, the current lack of efficacy data in certain populations (e.g., pediatrics, pregnant women, and autoimmune or immunocompromised individuals), and the emergence of more-transmissible viral variants all highlight the need for a large armamentarium of safe and effective COVID-19 vaccines (*4*, *5*).

The coronavirus efficacy (COVE) phase 3 trial (NCT04470427) of the mRNA-1273 COVID-19 vaccine, which is being conducted in the US in adults aged 18 and over, showed estimated vaccine efficacy against COVID-19 of 94% in the primary analysis (*6*). These efficacy data supported the FDA’s EUA of mRNA-1273 for the prevention of COVID-19 in adults (*7*). The mRNA-1273 vaccine has been shown to be highly effective in the elderly and in essential and frontline workers, including health care workers (*8*), and to have noninferior binding and neutralizing antibody responses in adolescents versus adults (*9*).

Correlates of protection, which are immunological markers that can be used to reliably predict the level of vaccine efficacy against a clinically relevant end point, such as COVID-19 (*10*–*12*), are highly sought in vaccine research. The identification and validation of a correlate of protection would expedite the clinical evaluation and regulatory approval process for existing vaccines for new populations, for vaccine regimen modifications, and for new vaccines. Neutralizing antibodies (nAbs) or binding antibodies (bAbs) have been established as a correlate of protection for vaccines against many viral diseases (*11*). The hypothesis that antibodies, whether elicited by infection or by spike protein–based vaccines, are a correlate of protection against COVID-19 is supported by diverse lines of evidence (*13*–*25*). For the mRNA-1273 vaccine, multiple severe acute respiratory syndrome coronavirus 2 (SARS-CoV-2) antibody markers—including immunoglobulin G (IgG) bAbs to the spike protein, IgG bAbs to the spike receptor-binding domain (RBD), and 50% inhibitory dilution (ID_50_) nAb titer—correlated with protection against SARS-CoV-2 replication after challenge in vaccinated rhesus macaques (*24*). Here, we assessed these same SARS-CoV-2 antibody markers as well as an 80% inhibitory dilution (ID_80_) nAb titer as correlates of risk of COVID-19 and as correlates of mRNA-1273 vaccine protection against COVID-19 in the COVE trial.

## Participant demographics

Table S1 describes demographics of the randomly sampled immunogenicity subcohort (*N* = 1010 vaccine, *N* = 137 placebo). Thirty-four percent of baseline SARS-CoV-2–negative per-protocol participants were age 65 or over, 40% were deemed to be at risk for severe COVID-19 illness (referred to as “at risk”), 47% were assigned female sex at birth, 32% were Hispanic or Latino, 46% were white and non-Hispanic, and 54% were from communities of color, with 18% Black or African American. Table S2 and figs. S1 and S2 describe the day 29 marker case-cohort set and the day 57 marker case-cohort set, which augment the immunogenicity subcohort with all vaccine breakthrough COVID-19 end point cases and make up the sets of participants included in the analyses of antibody markers measured at day 29 or day 57 as correlates, respectively.

## COVID-19 end points

Analyses of day 29 and day 57 antibody markers as correlates included vaccine breakthrough COVID-19 end points starting 7 days after day 29 (*n* = 46) and after day 57 (*n* = 36), respectively (fig. S3). Average follow-up of vaccine recipients was 116 days after day 29 and 88 days after day 57. All immune correlates analyses were prespecified, as detailed in the supplementary file Statistical Analysis Plan (SAP).

COVE follows participants for 2 years, which will enable future analyses of how the current level of antibodies correlates with instantaneous risk of COVID-19. Such analyses may inform how vaccine efficacy wanes as antibody levels wane and as new variants emerge, which in turn may inform decisions about the timing of a potential third dose of vaccination and/or the need to update vaccine composition (*26*).

## Antibody marker levels are lower in vaccine recipient cases versus noncases

At day 57, almost 100% of vaccine recipients had positive or detectable antibody response by all four markers (Table 1; table S3 shows assay limits for each marker). This was also true at day 29 for spike IgG and RBD IgG, whereas ID_50_ and ID_80_ titers were detectable in 82 and 64% of vaccine recipients, respectively. Each marker was moderately correlated between the day 29 and day 57 time points [Spearman rank correlation coefficient (*r*) = 0.53 to 0.62; fig. S4]. Together, the spike IgG and RBD IgG markers were tightly correlated (Spearman rank *r* = 0.94 and 0.97 at days 29 and 57, respectively; figs. S5 and S6) as were the ID_50_ and ID_80_ markers (*r* = 0.97 and 0.96 at days 29 and 57, respectively; figs. S5 and S6). Accordingly, some results focus on spike IgG and ID_50_. Each bAb marker was correlated with each neutralization marker at each time point (*r* = 0.73 to 0.80).

**Table 1.** Anti-spike and anti-RBD IgG response rates and geometric mean concentrations (GMCs) and pseudovirus neutralization titer ID_50_ and ID_80_ response rates and geometric mean titers (GMTs) by COVID-19 outcome status. Analysis based on baseline-negative per-protocol vaccine recipients in the day 29 marker or day 57 marker case-cohort sets. Median (interquartile range) number of days from dose one to day 29 was 28 (28 to 30) and from day 29 to day 57 was 28 (28 to 30). The *N* category under “Noncases in immunogenicity subcohort” indicates the number of noncases in the immunogenicity subcohort and hence with day 1, day 29, and day 57 antibody marker data, included in both the day 29 and day 57 marker correlates analyses. The *N* category under “COVID-19 cases” indicates either the number of vaccine breakthrough cases with day 1 and day 29 antibody marker data included (for day 29 marker analyses) or the number of vaccine breakthrough cases with day 1, day 29, and day 57 antibody data included (for day 57 marker analyses). See fig. S2. GM, geometric mean.

**Visit for** **marker**	**Marker**	COVID-19 cases*			**Noncases in immunogenicity subcohort**			**Comparison**	
		***N***	**Response rate** **(95% CI)**	**GMC or GMT** **(95% CI)**	***N***	**Response rate** **(95% CI)**	**GMC or GMT** **(95% CI)**	**Response rate** **difference** **(95% CI)**	**Ratio of GM** **(cases/noncases)** **(95% CI)**
Day 29	Anti-spike IgG (BAU/ml)	46	97.8% (85.4 to 99.7%)	183 (126 to 266)	1005	98.6% (97.4 to 99.2%)	318 (292 to 347)	−1% (−13 to 1%)	0.57 (0.39 to 0.84)
Day 29	Anti-RBD IgG (BAU/ml)	46	97.8% (85.4 to 99.7%)	207 (147 to 293)	1005	98.4% (97.2 to 99.1%)	327 (302 to 354)	−1% (−13 to 2%)	0.63 (0.44 to 0.90)
Day 29	Pseudovirus nAb ID_50_ (IU_50_/ml)	46	65.2% (50.1 to 77.8%)	7.6 (5.4 to 10.8)	1005	81.7% (78.8 to 84.3%)	13.0 (11.9 to 14.1)	−17% (−32 to −4%)	0.59 (0.41 to 0.84)
Day 29	Pseudovirus nAb ID_80_ (IU_80_/ml)	46	43.5% (29.7 to 58.4%)	18.0 (13.3 to 24.2)	1005	63.9% (60.4 to 67.3%)	29.0 (27.1 to 31.0)	−20% (−35 to −5%)	0.62 (0.46 to 0.84)
Day 57	Anti-spike IgG (BAU/ml)	36	100.0% (100.0 to 100.0%)	1890 (1449 to 2465)	1005	99.4% (98.2 to 99.8%)	2652 (2457 to 2863)	1% (0 to 2%)	0.71 (0.54 to 0.94)
Day 57	Anti-RBD IgG (BAU/ml)	36	100.0% (100.0 to 100.0%)	2744 (2056 to 3664)	1005	99.4% (98.3 to 99.8%)	3937 (3668 to 4227)	1% (0 to 2%)	0.70 (0.52 to 0.94)
Day 57	Pseudovirus nAb ID_50_ (IU_50_/ml)	36	100.0% (100.0 to 100.0%)	160 (117 to 220)	1005	98.7% (97.6 to 99.3%)	247 (231 to 264)	1% (1 to 2%)	0.65 (0.47 to 0.90)
Day 57	Pseudovirus nAb ID_80_ (IU_80_/ml)	36	97.2% (81.6 to 99.6%)	332 (248 to 444)	1005	98.3% (97.1 to 99.1%)	478 (450 to 508)	−1% (−17 to 2%)	0.69 (0.52 to 0.93)

*Cases for day 29 marker correlates analyses (intercurrent cases + post–day 57 cases) are baseline SARS-CoV-2–negative per-protocol vaccine recipients with the symptomatic infection COVID-19 primary end point diagnosed starting 7 days after day 29 through the end of the blinded phase. Cases for day 57 marker correlates analyses (post–day 57 cases) are baseline SARS-CoV-2–negative per-protocol vaccine recipients with the symptomatic infection COVID-19 primary end point diagnosed starting 7 days after day 57 through the end of the blinded phase. The last COVID-19 end point within the blinded phase occurred 100 days after day 57.

**Fig. 1.**
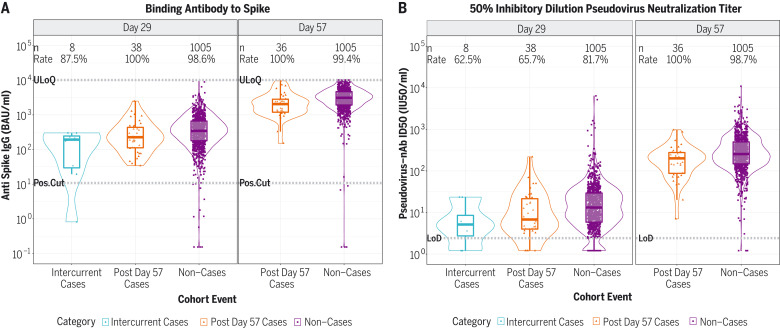
Anti-spike IgG concentration and pseudovirus neutralization ID_50_ titer by COVID-19 outcome status. (**A**) Anti-spike IgG concentration. (**B**) Pseudovirus neutralization ID50 titer. Data points are from baseline-negative per-protocol vaccine recipients in the day 29 marker or day 57 marker case-cohort set. The violin plots contain interior box plots with upper and lower horizontal edges representing the 25th and 75th percentiles of antibody level and middle line representing the 50th percentile. The vertical bars represent the distance from the 25th (or 75th) percentile of antibody level and the minimum (or maximum) antibody level within the 25th (or 75th) percentile of antibody level minus (or plus) 1.5 times the interquartile range. Each side shows a rotated probability density (estimated by a kernel density estimator with a default Gaussian kernel) of the data. Positive response rates were computed with inverse probability of sampling weighting. Pos.Cut, positivity cut-off; LoD, limit of detection; ULoQ, upper limit of quantitation. ULOQ = 10,919 for ID_50_ (above all data points). Positive response for spike IgG was defined by IgG > 10.8424 BAU/ml. Positive response for ID_50_ was defined by value > LOD (2.42). Post–day 57 cases are COVID-19 end points starting 7 days after day 57 through the end of the blinded follow-up (last COVID-19 end point was 126 days after dose 2); intercurrent cases are COVID-19 end points starting 7 days after day 29 through 6 days after day 57.

Figure 1 and fig. S7 show the day 29 and day 57 marker distributions by case or noncase status in vaccine recipients (fig. S8 in placebo recipients), and figs. S9 and S10 show marker values by participant age. For all eight markers, the geometric mean was lower in vaccine breakthrough cases than in vaccine recipient noncases, with geometric mean ratios (cases/noncases) and their 95% confidence interval (CI) upper bounds all <1 (Table 1).

Figures S11 and S12 show reverse cumulative distribution function curves of the eight markers, in the context of the overall vaccine efficacy estimates (*27*). Figure S13 shows the day 29 and/or day 57 marker values of vaccine breakthrough cases by timing of COVID-19 end point diagnosis.

## COVID-19 risk of vaccine recipients decreases as antibody marker levels increase

**Fig. 2.**
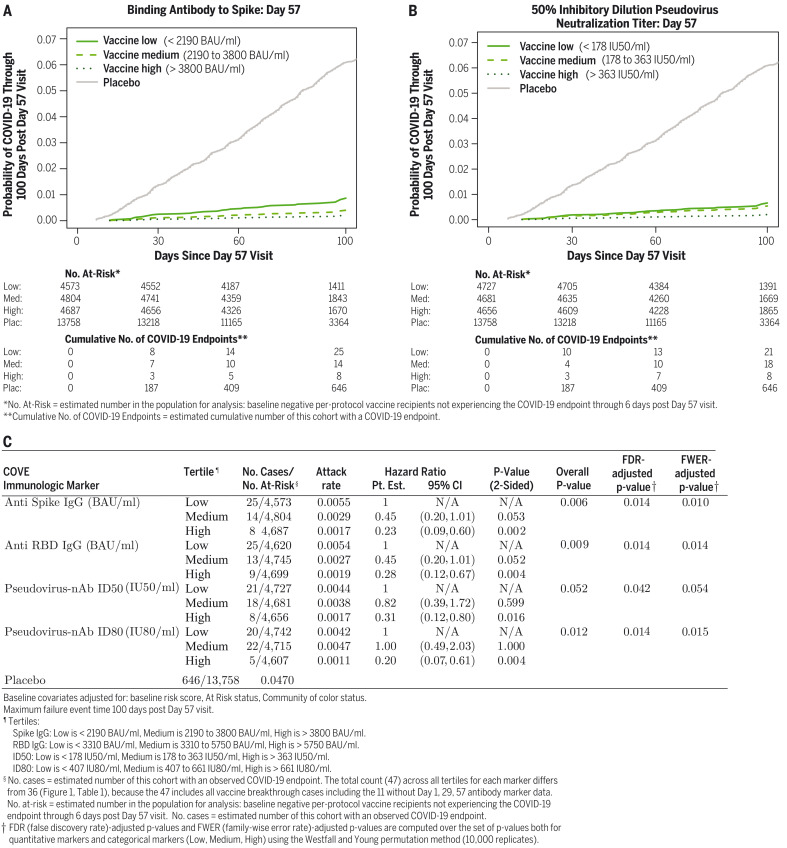
COVID-19 risk by antibody marker level. The plots and table show covariate-adjusted cumulative incidence of COVID-19 by low, medium, and high tertiles of day 57 IgG concentration or pseudovirus neutralization titer in baseline SARS-CoV-2–negative per-protocol participants. (**A**) Anti-spike IgG concentration. (**B**) ID_50_ titer. (**C**) IgG (spike and RBD) and pseudovirus neutralization titer (ID_50_ and ID_80_). The overall *P* value is from a generalized Wald test of whether the hazard rate of COVID-19 differed across the low, medium, and high subgroups. Baseline covariates are adjusted for baseline risk score, at risk status, and community of color status. Pt. Est., point estimate; FDR, false discovery rate; FWER, family-wise error rate.

**Fig. 3.**
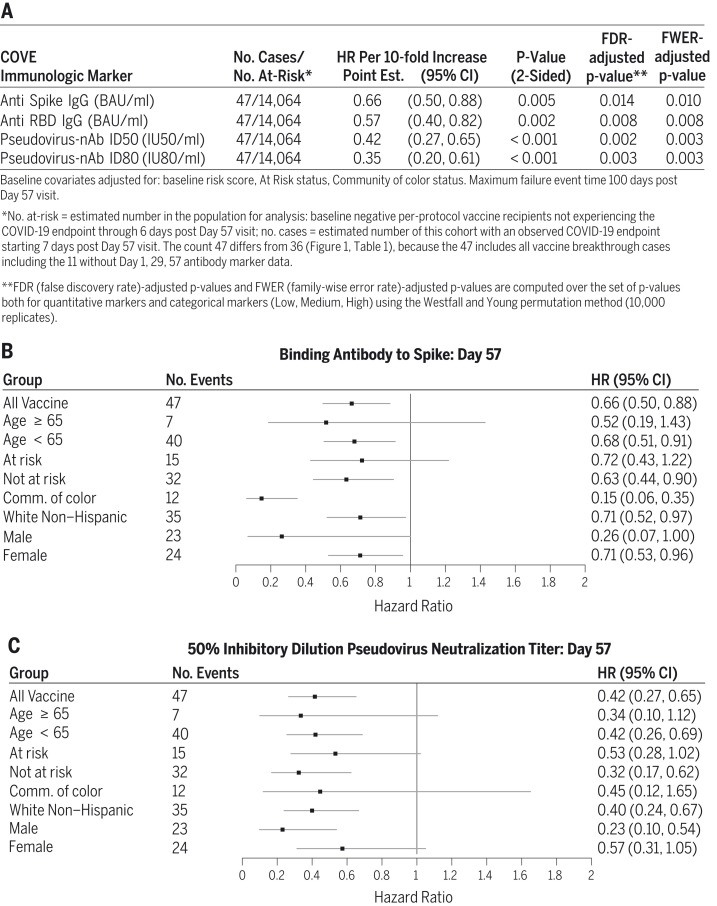
Hazard ratio of COVID-19 as antibody marker level increases. The table and plots show covariate-adjusted hazard ratios of COVID-19 per 10-fold increase in each day 57 antibody marker in baseline-negative per-protocol vaccine recipients overall and in subgroups. (**A**) Inferences for IgG (spike and RBD) and pseudovirus neutralization titer (ID_50_ and ID_80_). (**B**) Forest plots for spike IgG. (**C**) Forest plots for ID_50_. Baseline covariates are adjusted for baseline risk score, at risk status, and community (Comm.) of color status.

Figure 2 shows Cox model–based covariate-adjusted COVID-19 cumulative incidence curves for subgroups of vaccine recipients defined by tertile of day 57 IgG spike or ID_50_ (Fig. 2, A and B). Corresponding results for IgG RBD and ID_80_ are shown in fig. S14. (Details on covariate adjustment are given in the supplementary text, section S1; tables S4 to S7; and figs. S15 and S16.) Multiplicity-adjusted *P* values indicated significant inverse correlations with risk, with estimated hazard ratios for upper versus lower tertiles ranging between 0.20 and 0.31 (Fig. 2C). For quantitative day 57 markers, the estimated hazard ratio per 10-fold increase in marker value ranged between 0.35 and 0.66 (Fig. 3A), with multiplicity-adjusted *P* values indicating significant associations. Generally, similar results were obtained across prespecified vaccine recipient subgroups (Fig. 3, B and C, and fig. S17).

The four markers at day 29 were also significant inverse correlates of risk, with estimated hazard ratios for upper versus lower tertiles ranging between 0.19 and 0.32 (figs. S18 and S19) and estimated hazard ratios per 10-fold increase in marker value ranging between 0.19 and 0.54 (fig. S17). *P* values were smaller for day 29 markers than for day 57 markers, which indicates strengthened evidence for correlates of risk. If a day 29 immune marker in recipients of two mRNA-1273 doses becomes established as a correlate of protection, it could be a more practical surrogate marker than a day 57 marker. Notably, all participants in our correlates analysis received both dose 1 and dose 2, so the day 29 correlates results reflect the full effect of the two vaccine doses used in clinical practice.

**Fig. 4.**
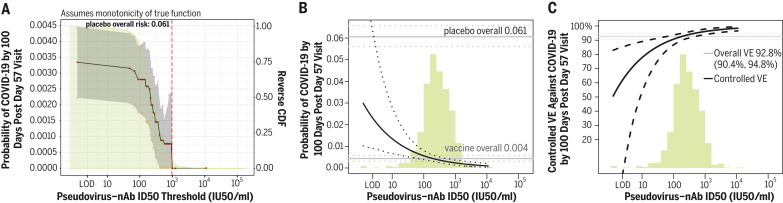
Further analyses of day 57 ID_50_ level as a correlate of risk and as a correlate of protection. (**A**) Covariate-adjusted cumulative incidence of COVID-19 by 100 days after day 57 by vaccinated baseline SARS-CoV-2–negative per-protocol subgroups defined by day 57 ID_50_ level above a threshold, with reverse cumulative distribution function (CDF) of day 57 ID_50_ level overlaid in green. The red dots are point estimates at 35 threshold values equally spaced over quantiles of the observed marker values, linearly interpolated by solid black lines. The gray shaded area is pointwise 95% CIs. The upper boundary of the green shaded area is the estimate of the reverse CDF of day 57 ID_50_ level in baseline SARS-CoV-2–negative per-protocol vaccine recipients. The vertical red dashed line is the day 57 ID_50_ threshold above which no post–day 57 COVID-19 end points occurred. (**B**) Covariate-adjusted cumulative incidence of COVID-19 by 100 days after day 57 by day 57 ID_50_ level. The dotted black lines indicate bootstrap pointwise 95% CIs. The upper and lower horizontal gray lines are the overall cumulative incidence of COVID-19 from 7 to 100 days after day 57 in placebo and vaccine recipients, respectively. (**C**) Vaccine efficacy (VE) (solid black line) by day 57 ID_50_ level, estimated using the method of Gilbert, Fong, and Carone (*28*). The dashed black lines indicate bootstrap pointwise 95% CIs. The horizontal gray line is the overall vaccine efficacy from 7 to 100 days after day 57, with the dotted gray lines indicating the 95% CIs [this number, 92.8%, differs from the 94.1% reported in (*6*), which was based on counting COVID-19 end points starting 14 days after day 29]. In (B) and (C), the green histograms are an estimate of the density of day 57 ID_50_ level in baseline-negative per-protocol vaccine recipients. Baseline covariates are adjusted for baseline risk score, at risk status, and community of color status.

The estimated cumulative incidence of COVID-19 by the end of blinded follow-up (100 days after day 57) for the entire vaccine group was 0.0033 (95% CI, 0.0022 to 0.0045). On the basis of nonparametric threshold regression, this cumulative incidence decreased across vaccinated subgroups with day 57 ID_50_ titer above a given threshold, with zero COVID-19 end points at ID_50_ titer above 1000 IU_50_/ml (Fig. 4A). The shape of cumulative incidence across threshold subgroups tracked the reverse cumulative distribution function of ID_50_ titer, which suggests a smooth incremental change in risk with titer (Fig. 4A). Based on the Cox model, Fig. 4B shows estimated cumulative incidence of COVID-19 by end of blinded follow-up across vaccinated subgroups with day 57 ID_50_ titer at specific titers, in contrast to Fig. 4A, which considers vaccinated subgroups with titers above specific values. For vaccine recipients with undetectable day 57 ID_50_ titer, estimated cumulative incidence was 0.030 (95% CI, 0.010 to 0.093), and it decreased to 0.014 (0.0067 to 0.028) at titer of 10, to 0.0056 (0.0039 to 0.0080) at titer of 100, and to 0.0023 (0.0013 to 0.0036) at titer of 1000 (Fig. 4B). The generalized additive model also supported inverse correlates of risk for all markers (figs. S20 and S21).

## Vaccine efficacy increases as antibody marker levels increase

Figure 4C shows titer-specific vaccine efficacy across day 57 ID_50_ titer levels, which, for a given titer level, is the estimated covariate-adjusted percent reduction in cumulative incidence of COVID-19 by the end of blinded follow-up as a result of vaccination generating the given titer level compared with being unvaccinated (*28*). Vaccine efficacy estimates increased with day 57 ID_50_ titer: At undetectable day 57 ID_50_, vaccine efficacy was 51% (95% CI, −51 to 83%), and at day 57 ID_50_ value of 10, 100, and 1000 IU_50_/ml, respectively, vaccine efficacy was 78% (54 to 89%), 91% (87 to 94%), and 96% (94 to 98%) (Fig. 4C). The increase in vaccine efficacy from 78 to 96% at ID_50_ values of 10 to 1000 IU_50_/ml, respectively, represents a 5.5-fold increase in vaccine-risk reduction (1 − vaccine efficacy = 22 versus 4%). Vaccine efficacy estimates also increased with day 29 ID_50_ neutralization titers: 79% (−62 to 90%), 93% (90 to 95%), 97% (95 to 99%), and 99% (97 to 100%) at the same IU_50_ per milliliter values (fig. S22).

Figures S23 to S28 show these results for the other six antibody markers. Conclusions for bAbs were similar to those for nAbs, with vaccine efficacy increasing with IgG levels, for example at day 57 spike IgG of 33, 300, and 4000 binding antibody units (BAU)/ml, vaccine efficacy was 85% (31 to 92%), 90% (77 to 94%), and 94% (91 to 96%), respectively. Another conclusion of these analyses is that subgroups with neutralization titer 10 IU_50_/ml (Fig. 4C) or with anti-spike IgG 33 BAU/ml (fig. S24C) have ~75 to 85% reduction in COVID-19 risk compared with being unvaccinated. Given the overall similarity of the bAb and nAb correlate of protection results, the potential value of the validated meso scale discovery (MSD) bAb assay for aiding vaccine approval decisions as a practical nonmechanistic correlate of protection (*12*) should be considered. This is because the MSD bAb assay is sensitive (table S3), robust, high-throughput, deployable, and easily standardized across viral strains, even though validated sensitive bAb detection may lack the specific immune function, such as neutralization.

## A sensitivity analysis further increases confidence that vaccine efficacy increases with antibody marker levels

A sensitivity analysis was conducted (supplementary text, section S2) assuming the existence of an unmeasured confounder associated with both the antibody marker and COVID-19 outcome that would make the estimated vaccine efficacy by marker curve flatter, with the specified amount of unmeasured confounding detailed in the SAP (section 12.1.2). The analysis indicated that vaccine efficacy estimates still increased with day 57 ID_50_ titer [90% (95% CI, 69 to 96%) at undetectable day 57 ID_50_ titer, 95% (93 to 97%) at day 57 ID_50_ titer of 500, and 96% (93 to 97%) at day 57 ID_50_ titer of 1000] (fig. S29C). A similar pattern of results occurred for all other nAb markers (fig. S29D and fig. S30, C and D). By contrast, estimated vaccine efficacy appeared to vary only minimally with each bAb marker when unmeasured confounding was assumed (fig. S29, A and B, and fig. S30, A and B). The sensitivity analysis based on E-values (*29*) of the vaccine recipient antibody tertile subgroups (supplementary text, section S2) supported the inference that vaccine efficacy was generally higher for the upper versus lower tertile subgroup (table S8), which suggests that vaccine efficacy would have still increased with each antibody marker level even if additional (hypothetical) unmeasured confounders had been present.

Given the overlap of marker distributions in vaccine breakthrough cases and vaccine recipient noncases (Fig. 1 and fig. S7), our results do not support that a day 29 or day 57 antibody marker could be highly effective in guiding individual decisions of whether to be revaccinated or boosted. However, if a vaccinated person has negative IgG response or undetectable neutralization response, on the basis of our results, it would be rational for this person to be concerned about relatively weak protection and to therefore prompt the seeking out of other means of protection.

## nAbs mediate about two-thirds of the mRNA-1273 vaccine efficacy

For bAbs at both time points, and for nAbs at day 57, a challenging issue is understanding vaccine efficacy for vaccine recipients with negative or undetectable antibody levels, given that <2% of vaccine recipients had negative or undetectable antibodies. Consequently, the 95% CIs about vaccine efficacy for these subgroups were wide, and assessment of mediation through these markers was not technically possible because of insufficient overlap of marker values in placebo and vaccine recipients. However, day 29 ID_50_ and ID_80_ titers could be assessed as mediators of vaccine efficacy by the Benkeser *et al*. method (*30*), given that 18 and 36% of vaccine recipients had undetectable titer, respectively, providing enhanced precision [e.g., estimated vaccine efficacy 79% (95% CI, 62 to 90%) at undetectable ID_50_]. The quantitative ID_50_ and ID_80_ variables were studied. An estimated 68.5% (58.5 to 78.4%) of vaccine efficacy was mediated by day 29 ID_50_ titer and 48.5% (34.5 to 62.4%) by day 29 ID_80_ titer (table S9).

This result of positive vaccine efficacy for the undetectable subgroup implies a lack of full mediation of vaccine efficacy through the day 29 antibody marker (*28*), with an estimated 68% of the overall vaccine efficacy mediated through day 29 ID_50_ titer. Therefore, if nAbs circulating on day 29 could be removed but the other consequences of vaccination remained, overall vaccine efficacy would be expected to be reduced by 68% (on the log scale), from 92 to 56%. However, because >98% of vaccine recipients achieved detectable nAbs by day 57, these day 29 mediation results do not reflect a complete deactivation of the nAb response to the level at both day 29 and day 57 (undetectable) that would have been obtained without vaccination. Yet, under the reasonable assumption that the vaccine’s effect on the risk of COVID-19 operating through the day 57 ID_50_ marker is nonnegative, 68% is a lower bound for the proportion of vaccine efficacy that is mediated through ID_50_ at both day 29 and day 57 (see conditions in supplementary text, section S2). In comparison, hemagglutination inhibition titer against the B/Brisbane/60/2008-like (Victoria lineage) strain of influenza virus (included in the trivalent inactivated influenza vaccine) mediated an estimated 57% of vaccine efficacy against virologically confirmed influenza B/Victoria illness (*31*). As hemagglutination inhibition titer has been used to guide influenza vaccine strain selection and approval, this defines a potential benchmark for influencing COVID-19 vaccine approval decisions (*32*).

A possible interpretation also consistent with our results is that neutralization as a biological function mediated a large proportion of the vaccine efficacy, but the specific day 29 ID_50_ and ID_80_ immune markers studied—measured with a particular immunoassay—had inadequate sensitivity to quantify low-level neutralization below the positivity cutoff that could be present and functionally important. Passive transfer of purified IgG from mRNA-1273–immunized rhesus macaques protected golden Syrian hamsters from disease after SARS-CoV-2 challenge, which suggests that functionally active antibodies can mediate protection (*24*). However, additional immune markers are likely needed to fully explain the observed vaccine efficacy in COVE—for example, markers measuring additional immune functions beyond neutralization (e.g., Fc effector functions or functional T cells), markers not measured fully in serum (e.g., mucosal), and/or anamnestic responses not fully represented by a single time point measurement.

Further clarification of functional mediation of protection may be provided by future correlates analyses that study antibody markers over time in relation to the timing of breakthrough infections of variants with variable neutralization sensitivity, with the antibodies measured against the variants of concern as well as against the ancestral strain. In particular, future research in COVE aims to measure bAbs and nAbs to the Delta variant in the same immunogenicity subcohort as examined in the current study and in all additional vaccine breakthrough cases that occur during follow-up. This should enable analyses to assess the consistency of an ancestral-strain correlate of protection for ancestral-strain COVID-19 compared with a Delta-variant correlate of protection against Delta-variant COVID-19.

## Similar results are seen in a cross-trial–cross-platform comparison

Our use of validated assays, with all results reported in WHO international units (IU) or calibrated to WHO international standards, enables comparison with other studies and vaccine platforms. Immune correlates results for the COV002 trial (*33*), which is testing the AZD1222 chimpanzee adenoviral-vectored vaccine (also called ChAdOx1 nCoV-19), are available (*19*). The COV002 correlates results for spike IgG and RBD IgG can be quantitatively compared with the COVE results by virtue of the same MSD assay platform, conversion of IgG concentration to WHO international units per milliliter, and the same antibody measurement time—4 weeks after the second dose. Estimated AZD1222 vaccine efficacy was 70 and 90% at spike IgG levels of 113 [95% CI < limit of detection (LOD) = 0.31 to 245] and 899 (369 to NC) BAU/ml, respectively, and at RBD IgG levels of 165 (<LOD = 1.59 to 452) and 2360 (723 to NC) BAU/ml, respectively (where NC means not calculated) (*19*). For COVE, there is low precision at 70% vaccine efficacy because few vaccine recipients had IgG < 100 BAU/ml, such that we only compare results at 90% vaccine efficacy. Estimated mRNA-1273 vaccine efficacy was 90% at day 57 spike IgG level 298 (1 to 1786) BAU/ml and at day 57 RBD IgG level of 775 (29 to 2819) BAU/ml. Although the point estimates of IgG levels at 90% efficacy were about three times as high for COV002 compared with those for COVE, the overlapping CIs are consistent with similar results across the two trials.

Pseudovirus neutralization results can also be compared between the trials using ID_50_ titers calibrated to the international standard, where estimated AZD1222 vaccine efficacy was 70 and 90% at ID_50_ titer of 8 (<LOD = 2.42 to 26) and 140 (43 to NC) IU_50_/ml, compared with COVE results at ID_50_ titer of 4 (<LOD = 2.42 to 22) and 83 (16 to 188) IU_50_/ml. These results support that nAb titers have a similar quantitative relationship with vaccine efficacy for the two vaccine platforms, which is promising for potential applications of a neutralization biomarker. The materials and methods provide a sensitivity analysis comparing correlate of protection results between COV002 and COVE.

With the caveats of different study end points and hosts, the COVE results are also consistent with results on spike IgG and nAb titers as correlates of protection against SARS-CoV-2 replication in mRNA-1273–vaccinated rhesus macaques. For instance, all macaques with spike IgG > 336 IU/ml at 4 weeks after second dose were protected from >10,000 subgenomic RNA copies per milliliter in bronchoalveolar lavages (*24*), and in COVE, day 57 spike IgG of 336 IU/ml corresponded to 90% vaccine efficacy against COVID-19 (fig. S24).

## Conclusions

Our findings that all evaluated bAb and nAb markers strongly inversely correlated with COVID-19 risk and directly correlated with vaccine efficacy add evidence toward establishing an immune marker surrogate end point for mRNA COVID-19 vaccines. Moreover, the prespecification of the analyses and the absence of post hoc modifications bolsters the credibility of our conclusions.

For per-protocol recipients of two doses of mRNA-1273 COVID-19 vaccine in the COVE clinical trial, all four antibody markers at day 29 and at day 57 were inverse correlates of risk of COVID-19 occurrence through ~4 months after the second dose. Based on any of the antibody markers, estimated COVID-19 risk was about 10 times as high for vaccine recipients with negative or undetectable values compared with the estimated risk for those with antibodies in the top 10% of values. The nonparametric threshold analyses (Fig. 4A) suggested a continuum model where COVID-19 risk decreased incrementally with increasing increments in antibody level rather than a threshold model where an antibody cut-point sharply discriminated risk.

Together with evidence from other studies, the current results support that neutralization titer is a potential surrogate marker for mRNA-1273 vaccination against COVID-19 that can be considered as a primary end point for basing certain provisional approval decisions. For example, an immunogenicity noninferiority approach has been proposed for adding vaccine spike variants and boosters (*34*). An advantage of a noninferiority approach is avoiding the need to specify an absolute antibody benchmark for approval, such as one based on the percentage of vaccine recipients with ID_50_ titer above a threshold and geometric mean titer above a threshold. However, some applications may be aided by an absolute benchmark if data allowing head-to-head noninferiority evaluation are unavailable. Such a benchmark based on ID_50_ values from vaccinated individuals in a bridging study could be based on predicted vaccine efficacy being sufficiently high, where, for example, predicted vaccine efficacy could be calculated on the basis of the COVE correlates of protection results (Fig. 4C) and averaging over the distribution of ID_50_ values.

The evidence level for justifying various bridging applications differs across applications. Currently, confidence is greatest for bridging short-term vaccine efficacy (i.e., over 4 to 6 months) against COVID-19 to new subgroups for the same vaccine (e.g., to young children) or for bridging to a modified dose or schedule for the same vaccine (e.g., completing the primary series with a third dose). Less evidence is available to buttress the use of a humoral immune marker to predict long-term protection, to bridge to a new vaccine within the same vaccine platform, or to bridge to new spike variant inserts for the same vaccine. An open question challenging the latter application is whether higher nAb responses to emergent SARS-CoV-2 variants, such as Delta, will be needed to achieve similar levels of vaccine efficacy, although modeling data are beginning to support the ability to make cross-variant predictions (*16*). Less evidence still is available for justifying bridging to a new candidate vaccine in a different vaccine platform. When immune correlates results are available from several COVID-19 phase 3 vaccine efficacy trials covering a multiplicity of vaccine platforms, it will be possible to conduct validation analyses of how well antibody markers can be used to predict vaccine efficacy across platforms (*35*). Uncertainties in bridging predictions can also be addressed by animal models that characterize immunological mechanisms of vaccine protection and by postauthorization or postapproval vaccine effectiveness studies (*36*). Notably, immune marker–based provisional approval mechanisms require postapproval studies verifying that the vaccine provides direct clinical benefit, such that the rigorous design and analysis of such studies is a critical component of the decision-making process for use of immune markers to accelerate the approval and distribution of vaccines.

Limitations of this immune correlates study include the inability to control for SARS-CoV-2 exposure factors (e.g., virus magnitude) and a lack of experimental assignment of antibody levels, which implies that the study could evaluate statistical correlates of protection or surrogate end points but not mechanistic correlates of protection (*10*). Additionally, scope limitations include the following: (i) the lack of data for assessing correlates against other outcomes besides COVID-19 (e.g., severe COVID-19, asymptomatic SARS-CoV-2 infection, infection regardless of symptomology, and viral shedding); (ii) the lack of assessment of non–antibody-based correlates (e.g., spike-specific functional T cell responses, which were not feasible to assess in the context of this study); (iii) the relatively short follow-up time of 4 months that precluded the assessment of immune correlate durability; (iv) the relatively small number of COVID-19 cases; (v) the lack of assessment of correlates for recipients of only one mRNA-1273 dose; (vi) the inability to assess the effects of boosting (homologous or heterologous) because this study pre-dated the addition of a third dose; (vii) the lack of data for assessing the potential contribution of anamnestic responses to the immune correlates; and (viii) the fact that almost all COVID-19 cases resulted from infections with viruses with a spike sequence similar to that of the vaccine strain, which precluded the assessment of robustness of correlates to SARS-CoV-2 variants of concern. However, the relative uniformity in circulating virus is also a strength in affording a clear interpretation as correlates against COVID-19 caused by variants genetically close to the vaccine. An additional strength is the racial and ethnic diversity of the trial participants and the large number of diverse participants sampled for immunogenicity measurements (*37*).

## Supplementary Material

20211123-1

## Supplementary Materials

### This PDF file includes:

Materials and Methods Supplementary Text Figs. S1 to S30 Tables S1 to S9 Immune Assays Team Collaborator List Moderna, Inc. Team Collaborator List CoVPN/COVE Team Collaborator List USG/CoVPN Biostatistics Team Collaborator List References (*39*–*53*)

### Other Supplementary Material for this manuscript includes the following:

MDAR Reproducibility Checklist

MDAR Reproducibility Checklist

## Notes

View/request a protocol for this paper from *Bio-protocol*.
