# Supplementary material for: Immune correlates analysis of the mRNA-1273 COVID-19 vaccine efficacy clinical trial
Source: Science. 2021 Nov 23;375(6576):43–50. doi: 10.1126/science.abm3425 (PMC9017870; doi:10.1126/science.abm3425)
Supplement: Supplementary file 3 — MDAR Reproducibility Checklist [file science.abm3425_mdar_reproducibility_checklist.pdf]

Materials Design Analysis Reporting (MDAR)
Checklist for Authors

The MDAR framework establishes a minimum set of requirements in transparent reporting applicable to studies in the life sciences (see Statement of Task: doi:10.31222/osf.io/9sm4x.). The MDAR checklist is a tool for authors, editors, and others seeking to adopt the MDAR framework for transparent reporting in manuscripts and other outputs. Please refer to the MDAR Elaboration Document for additional context for the MDAR framework.

For all that apply, please note where in the manuscript the required information is provided.

Materials:

Newly created materials	indicate where provided: page no/section/legend)	n/a
The manuscript includes a dedicated "materials availability statement" providing transparent disclosure about availability of newly created materials including details on how materials can be accessed and describing any restrictions on access.	Page 29, lines 16-19	

Antibodies	indicate where provided: page no/section/legend)	n/a
For commercial reagents, provide supplier name, catalogue number and RRID, if available.	96-well flat-bottom poly-L-lysine-coated culture plates (Corning, cat. no. 354516) (page 24, supplementary material)<br>Promega 1X lysis buffer (Promega, cat. no. E1531) and Bright-Glo luciferase reagent (Promega, cat. no. E2650) (page 24, supplementary material)<br>National Institute for Biological Standards and Controls (NIBSC): WHO International Standard for Anti-SARS-CoV-2 Immunoglobulin (20/136) (page 25, supplementary material)<br>sterile distilled water (Invitrogen, Cat No. 10977015, Ultra-pure DNase, RNase free, Lot 2186762, Exp 30-Aug-2022) (page 25, supplementary material)	

DNA and RNA sequences	indicate where provided: page no/section/legend)	n/a
**Short novel DNA or RNA including primers, probes:** Sequences should be included or deposited in a public repository.		X

Cell materials	indicate where provided: page no/section/legend	n/a
**Cell lines:** Provide species information, strain. Provide accession number in repository **OR** supplier name, catalog number, clone number, **OR** RRID.	293T/ACE2 cells: “293T/ACE2 cells (293T cell line stably overexpressing the human ACE2 cell surface receptor protein, obtained from Drs. Mike Farzan and Huihui Mu at Scripps)” top of page 24, supplementary material<br>293T cells: “(human embryonic kidney cells in origin; obtained from American Type Culture Collection, cat. no. CRL-11268)” top of page 24, supplementary material	
**Primary cultures:** Provide species, strain, sex of origin, genetic modification status.		X

Experimental animals	indicate where provided: page no/section/legend)	n/a
**Laboratory animals or Model organisms:** Provide species, strain, sex, age, genetic modification status. Provide accession number in repository **OR** supplier name, catalog number, clone number, **OR** RRID.		X
**Animal observed in or captured from the field:** Provide species, sex, and age where possible.		X

Plants and microbes	indicate where provided: page no/section/legend)	n/a
**Plants:** provide species and strain, ecotype and cultivar where relevant, unique accession number if available, and source (including location for collected wild specimens).		X

**Microbes:** provide species and strain, unique accession number if available, and source.		X

Human research participants	indicate where provided: page no/section/legend) or state if these demographics were not collected	n/a
If collected and within the bounds of privacy constraints report on age, sex and gender or ethnicity for all study participants.	"Table S1 describes demographics of the randomly sampled immunogenicity subcohort (N = 1010 vaccine, N = 137 placebo)." (line 12 page 5)	

Design:

Study protocol	indicate where provided: page no/section/legend)	n/a
If study protocol has been pre-registered, provide DOI. For clinical trials, provide the trial registration number **OR** cite DOI.	"The Coronavirus Efficacy (COVE) phase 3 trial (NCT04470427) of the mRNA-1273 COVID-19 vaccine…" (line 9 page 4)	

Laboratory protocol	indicate where provided: page no/section/legend)	n/a
Provide DOI **OR** other citation details if detailed step-by-step protocols are available.	Neutralization assay: Shen et al. (38) is referenced ("Neutralization assay" subheading of "Laboratory methods" in "Materials and Methods"in the supplement).	

Experimental study design (statistics details)		
**For in vivo studies:** State whether and how the following have been done	**indicate where provided: page no/section/legend. If it could have been done, but was not, write not done**	**n/a**
Sample size determination	Section 5.2 in the Statistical Analysis Plan (provided in the supplementary material) describes how the Stage 1 immune correlates analysis of Day 29 and Day 57 antibody markers (reported in this manuscript) is performed after reaching a minimum of 25 evaluable breakthrough COVID-19 endpoint cases in the vaccine arm. This randomized, placebo-controlled phase 3 vaccine efficacy trial had a sample size of about 30,000 volunteers (15,000 assigned to the vaccine arm and 15,000 assigned to the placebo arm), with sample size selected such that there would be at least 90% statistical power to meet WHO/FDA success criteria for vaccine efficacy against the COVID-19 primary endpoint (point estimate exceeding 50% and lower 95% confidence limit exceeding 30%) if the true vaccine efficacy against COVID-19 is at least 60%.	X
Randomisation		X
Blinding		X
Inclusion/exclusion criteria		X

Sample definition and in-laboratory replication	indicate where provided: page no/section/legend	n/a
State number of times the experiment was replicated in laboratory.	"The assays were performed between January 5, 2021 and April 16, 2021 by four operators. All assays used either a 1:20 or 1:30 start dilution and a 5-fold dilution series for a total of 8 dilutions. One vial of the standard was reconstituted on January 5 and assayed once on each of 10 plates in a single setting by a single operator (EY) on January 5, 2021; the plates were read on January 8, 2021 (Exp ID EY18-134 in Table 1). These 10 assay results were described in Duke-02-MVR-COVID0001.2. In response to FDA/CBER recommendations (MF 026862, comments dated March 1, 2021), additional assays were performed over different operators and days to yield more precise estimates of the calibration factors. Additional vials of the reagent were reconstituted on March 20, 2021 and assayed in quadruplicate by two operators on two days, corresponding to technical replicates. A third operator assayed the reconstituted standard in quadruplicate on 3 days. All assays were set up within 24 days of reconstitution." Page 25, supplementary material	

Define whether data describe technical or biological replicates.	"One set of eight wells received cells + virus (virus control) and another set of eight wells received cells only (background control), corresponding to technical replicates." Page 24, supplementary material "Additional vials of the reagent were reconstituted on March 20, 2021 and assayed in quadruplicate by two operators on two days, corresponding to technical replicates." Page 25, supplementary material	

Ethics	indicate where provided: page no/section/legend	n/a
**Studies involving human participants:** State details of authority granting ethics approval (IRB or equivalent committee(s), provide reference number for approval.	"The Institutional IRB approval number for the use of human sera in the neutralization assay as described here is Pro00105358 (DUHS Institutional Review Board, 2424 Erwin Rd, Durham, NC, 919.668.5111, Federalwide Assurance No: FWA 00009025Suite 405)." Page 24, supplementary material	
**Studies involving experimental animals:** State details of authority granting ethics approval (IRB or equivalent committee(s), provide reference number for approval.		X
**Studies involving specimen and field samples:** State if relevant permits obtained, provide details of authority approving study; if none were required, explain why.		X

Dual Use Research of Concern (DURC)	indicate where provided: page no/section/legend	n/a
If study is subject to dual use research of concern regulations, state the authority granting approval and reference number for the regulatory approval.		X

Analysis:

Attrition	indicate where provided: page no/section/legend	n/a
Describe whether exclusion criteria were preestablished. Report if sample or data points were omitted from analysis. If yes report if this was due to attrition or intentional exclusion and provide justification.		X

Statistics	indicate where provided: page no/section/legend	n/a
Describe statistical tests used and justify choice of tests.	In Fig. 2 legend: "The overall p-value is from a generalized Wald test of whether the hazard rate of COVID-19 differed across the Low, Medium, and High subgroups."<br>In Fig. 3A: "FDR (false discovery rate)-adjusted p values and FWER (family-wise error rate)-adjusted p values are computed over the set of – values both for quantitative and categorical markers (Low, Medium, High) using the Westfall and Young permutation method (10,000 replicates)."<br>See also "Hypothesis testing" in "Materials and methods" (supplementary material): "All p-values were two-sided. For each set of hypothesis tests for Day 29 and Day 57 marker correlates of risk separately, Westfall-Young multiplicity adjustment (46) was applied to obtain false-discovery rate adjusted p-values and family-wise error rate adjusted p-values" and page 27, supplementary material: "Geometric mean concentration (GMC) or titer (GMT) and their ratios between cases vs. non-cases were estimated with 95% CIs based on the t-distribution with IPS weighting."<br><br>Use of pre-specified family-wise error rate multiple hypothesis testing adjustment was needed for inferring that findings on correlates are robust to potential false positives. The Westfall-Young method was selected because it can provide improved power compared to approaches that do not account for the correlation in the set of test statistics. The commonly used Holm-Bonferroni method (which ignores correlations of test statistics) gave similar results as the Westfall-Young method (results not shown).	

Data availability	indicate where provided: page no/section/legend	n/a
For newly created and reused datasets, the manuscript includes a data availability statement that provides details for access or notes restrictions on access.	"Data and materials availability: As the trial is ongoing, access to participant-level data and supporting clinical documents with qualified external researchers may be available upon request and subject to review once the trial is complete. The code is publicly available at https://github.com/CoVPN/correlates_reporting_usgcove_archive." (page 29)	X
If newly created datasets are publicly available, provide accession number in repository **OR** DOI **OR** URL and licensing details where available.		X
If reused data is publicly available provide accession number in repository **OR** DOI **OR** URL, **OR** citation.		X

Code availability	indicate where provided: page no/section/legend	n/a

For all newly generated custom computer code/software/mathematical algorithm or re-used code essential for replicating the main findings of the study, the manuscript includes a data availability statement that provides details for access or notes restrictions.	In the data availability statement (Page 29): "The code is publicly available at https://github.com/CoVPN/correlates_reporting_usgcove_archive."	
If newly generated code is publicly available, provide accession number in repository, **OR** DOI **OR** URL and licensing details where available. State any restrictions on code availability or accessibility.	In the data availability statement (Page 29): "The code is publicly available at https://github.com/CoVPN/correlates_reporting_usgcove_archive."	
If reused code is publicly available provide accession number in repository **OR** DOI **OR** URL, **OR** citation.		X

Reporting

MDAR framework recommends adoption of discipline-specific guidelines, established and endorsed through community initiatives. Journals have their own policy about requiring specific guidelines and recommendations to complement MDAR.

**Adherence to community standards**	**indicate where provided: page no/section/legend**	**n/a**
State if relevant guidelines (e.g., ICMJE, MIBBI, ARRIVE) have been followed, and whether a checklist (e.g., CONSORT, PRISMA, ARRIVE) is provided with the manuscript.		X
